# Measuring the effects of community polygyny on intimate partner violence: a multilevel modeling using nationally representative cross-sectional data

**DOI:** 10.1186/s12978-025-02037-7

**Published:** 2025-05-29

**Authors:** Zacharie Tsala Dimbuene, Bright Opoku Ahinkorah, Dickson Abanimi Amugsi

**Affiliations:** 1 School of Population and Development Sciences, University of Kinshasa, Kinshasa, Democratic Republic of the Congo; 2 School of Public Health, Faculty of Health, University of Technology Sydney, Sydney, Australia; 3 African Population and Health Research Center (APHRC), Nairobi, Kenya

## Abstract

**Background:**

Intimate partner violence (IPV) has received increasing attention the last three decades and it has been recognized as health, social, and human right issues across the world. Worldwide, sub-Saharan Africa is the most affected region. In the search of putative factors associated with IPV, women in polygamous marriages were found to be more exposed to IPV compared with those in monogamous marriages. However, previous research focused on polygyny at individual level; therefore, ignoring possible heterogeneity of the “acceptance of polygyny” across communities. This paper developed the concept of “*community polygyny*” in Central Africa and tested its associations with IPV. Furthermore, the paper tested interactions between the community polygyny and (*i*) polygyny at women’s level; (*ii*) women’s education; and (*iii*) urban residence.

**Methods:**

The paper used recent Demographic and Health Surveys of four countries in Central Africa (Democratic Republic of the Congo, Cameroon, Gabon, and Chad). Multilevel binary logistic regression analyses (additive and multiplicative models) were carried out. Findings were reported as adjusted odds ratios (aOR) at 95% Confidence Interval (95%CI).

**Main findings:**

Findings indicated an inverse-relationship between the percentage of women living in polygamous marriages and IPV. Indeed, the percentage of polygamous marriages was higher in Chad (34.3%) compared with the Democratic Republic of the Congo (18.6%), Cameroon (14.6%), and Gabon (13.9%). However, the percentage of women who experienced intimate partner violence in the last 12 months was lower in Chad (18.9%) compared with the Democratic Republic of the Congo (43.5%), Gabon (40.3%), and Cameroon (30.8%). In multivariate results, findings showed that a sizeable percentage of intraclass correlation (ICC) of IPV in the selected countries was explained at cluster level: ICC = 18.2% [95%CI: 15.0%-21.9%]; ICC = 14.3% [95%CI: ICC = 10.9%-18.5%]; ICC = 7.8% [95%CI: 5.3%-11.3%]; and ICC = 29.5% [95%CI: 23.7%-35.9%] in the Democratic Republic of the Congo, Cameroon, Gabon and Chad, respectively. Community polygyny (from Model 2) showed different patterns. In the DRC, it was positively and significantly associated with the likelihood of IPV (aOR = 2.890; 95%CI: 1.461–5.719). In contrast, it showed a negative association with IPV in Cameroon (aOR = 0.278; 95%CI = 0.143–0.539); in Gabon (aOR = 0.504; 95%CI = 0.237–1.074); and Chad (aOR = 0.749; 95%CI = 0.319–1.763).

**Conclusion:**

Findings from this study substantiates the importance of moving beyond individuals’ characteristics and incorporating the collective mindset of polygyny to fully capture the effects of polygyny on intimate partner violence in Central Africa. Previous research pointed out the negative effects of polygyny at individual level. This study showed that community polygyny, since communities might differ on the acceptance levels towards polygyny, is equally important to understand how polygyny could affect the prevalence of intimate partner violence in Central Africa. Therefore, interventions aimed at eliminating intimate partner violence should integrate communities’ influences on intimate partner violence in Central Africa and worldwide.

**Supplementary Information:**

The online version contains supplementary material available at 10.1186/s12978-025-02037-7.

## Introduction

Intimate partner violence (IPV) is a major global public health and human rights problem that disproportionally affects women and minority populations [1–6]. It is estimated that approximately 736 million women worldwide have experienced lifetime IPV, and this varies by countries and regions [6]. For instance, while sub-Saharan Africa (SSA) is the most affected region around the world, Central Africa has the highest prevalence of IPV for both lifetime (44%) and past year IPV (32%) experience compared with other sub-regions in SSA. Furthermore, previous studies have extensively documented a wide range of negative health outcomes of IPV on women and girls, including stillbirths and miscarriages, and sexually transmitted infections (STIs) [7]. Likewise, women who experienced IPV were more likely to suffer from depression, post-traumatic stress disorder, psychological distress, and suicidal thoughts [8–10].

The high prevalence of IPV attracted the attention of the international community. For example, the Sustainable Development Goals (SDGs), goal 5 target 5.2 sets out to reduce violence against girls and women, including IPV across countries [11]. Commendable efforts have been made in the last two decades to identify risk factors (e.g., poverty, lower education, attitudes towards violence, controlling behaviours, alcohol use, young age, experiencing childhood abuse or witnessing domestic violence, outside sexual relationships, among others) associated with IPV using different theoretical approaches and frameworks [1, 12] including feminism [12–14], cultural spillover theory [15], the social ecological model [16, 17], conflict of interest [18], and the intersectionality [19, 20], among others. In this search, researchers identified risk factors associated with IPV at community, sub-national and national levels [21, 22].

This paper focuses on polygamy (or polygyny) which is most common in a region known as the “polygamy belt” in West Africa and Central Africa where countries (e.g., Burkina Faso, Mali, Niger, Nigeria, Tanzania) have the highest polygamy prevalence in spite of its decline [23, 24]. In the last two decades, efforts have been made to unpack the effects of polygyny on IPV [25–29]. In these efforts however, polygyny has always been analyzed at individual level. Although there might be justifiable reasons (e.g., economic, emotional, cultural motives. See [30, 31] for more details) why people chose to engage in polygamous marriages, this paper posits that communities do vary in terms of the polygyny “acceptance”, whether people collectively endorse or reject polygamy. Indeed, polygamy in sub-Saharan Africa (SSA) is not only a type of marriage but also a value system, which is socially acceptable for several reasons such as economic security, increased social prestige and power, satisfaction of the desire for large families, and somehow a solution for infertile women [32]. As such, the effects of polygyny on IPV might vary across communities contingent on the prevalence of polygyny within communities. This paper developed the concept of “*community polygyny*”, the share of women living in polygynous unions within a community and tests its association with IPV in four countries in Central Africa (Cameroon, Chad, Democratic Republic of the Congo, and Gabon) using multilevel binary logistic modeling. Moreover, the paper tested three interactions which, to the best of our knowledge, have never been tested in previous research: community polygyny with (*i*) polygynous unions; (*ii*) women’s education, and (*iii*) urban residence.

### Methods

#### Study setting

Central Africa offers a unique case to study the cultural roots of polygyny on IPV two main reasons. First, Central Africa has the worst indicators on IPV as aforementioned. Indeed, previous research reported that IPV prevalence was higher in Central Africa compared with other regions in SSA [33, 34]. Yet, Central Africa is not the most affected area in terms of polygyny prevalence [35], even though the region still is a mix of traditional patrilineal and matrilineal societies [36], despite the increasing urbanization observed in the region over the last three decades [37]. This essential feature is important to understand the geographical distribution of IPV in the region since a set of theories (e.g., feminist perspective) explain IPV as a consequence of patriarchy, the male dominance over women in many societies around the world [38–40]. Second, previous research highlighted the economic costs of IPV worldwide [41, 42]. Moreover, studies showed that Central Africa exhibited the worst socio-economic indicators (e.g., corruption index; democratic accountability; law and order) [43]. As such, IPV might be seen as a double burden for socio-economically affected settings.

#### Data

The paper analyzed the most recent Demographic and Health Surveys (DHSs) data from Cameroon, Chad, the Democratic Republic of the Congo (DRC), and Gabon. DHSs are high quality nationally representative data collected in low- and middle-income countries (LMICs). The DHS uses a two-stage sampling design. The first stage involves the selection of sample points or clusters from an updated master sampling frame constructed in accordance with country’s administrative divisions or domains. These domains are further stratified into urban and rural areas. From the urban areas, neighbourhoods are sampled from cities and towns whereas villages and chiefdoms are sampled for rural areas. The clusters are selected using systematic sampling with probability proportional to size. Household listing is then conducted in all the selected clusters to provide a complete sampling frame for the second stage selection. The second stage of selection involves a systematic sampling of the households listed in each cluster, and households to be included in the survey are then randomly selected. The rationale for the second stage selection is to ensure adequate sample to estimate the key indicators with acceptable precision. All men and women aged 15–59 and 15–49, respectively, in the selected households were eligible to participate in the survey if they were either usual residents of the household or visitors present in the household on the night before the survey. Analyses in this paper were restricted to married and cohabiting women.

#### Variables measurement

##### Outcome

The outcome variable of interest was intimate partner violence (IPV), including physical, emotional, and sexual violence [26, 44]. The sub-components of IPV were derived from the domestic violence module. In this optional module, questions about domestic violence in the last 12 months were asked, based on a modified version of the conflict tactics scale [18, 45]. Questions for each sub-component and responses are summarized in Table 1 below.

**Table 1:** Questions and responses about intimate partner violence

Components of IPV and questions	Responses	Operational definition
Physical violence (7 items)		
1. Husband ever pushed, shook, or threw something 2. Husband slapped 3. Husband punched her with his fist or something harmful 4. Husband kicked or dragged 5. Husband strangled or burnt 6. Husband threatened her with a knife, gun, or other weapons 7. Husband twisted arm or pulled hair	Responses included 0 “Never”; 1 “Often”; 2 “Sometimes”; and 3 “Yes, but not in the last 12 months”	The item was recorded 0 “No” if wife reported “Never” or “Yes, but not in the last 12 months” and 1 if wife reported “Often” or “Sometimes”. The new variable ranged from 0 to 7
Emotional violence (3 items)		
1. Husband humiliated 2. Husband threatened to harm 3. Husband insulted or made feel bad	Responses included 0 “Never”; 1 “Often”; 2 “Sometimes”; and 3 “Yes, but not in the last 12 months”	The item was recorded 0 “No” if wife reported “Never” or “Yes, but not in the last 12 months” and 1 if wife reported “Often” or “Sometimes”. The new variable ranged from 0 to 3
Sexual violence (3 items)		
1. Husband ever physically forced wife into unwanted sex 2. Husband ever forced wife into other unwanted sexual acts 3. Respondent has been physically forced to perform sexual acts she didn’t want to	Responses included 0 “Never”; 1 “Often”; 2 “Sometimes”; and 3 “Yes, but not in the last 12 months”	The item was recorded 0 “No” if wife reported “Never” or “Yes, but not in the last 12 months” and 1 if wife reported “Often” or “Sometimes”. The new variable ranged from 0 to 3

###### Key independent variables

This study is interested in the associations between polygyny and IPV at individual and community levels. Women who reported that their husbands had no other wives were considered being in monogamous marriages, while those who indicated that their husbands had at least one or more other wives were considered living in polygamous marriages. Therefore, the variable ‘polygyny’ at individual level is a dichotomous variable taking the value “1” if the woman was living in polygamous unions and “0” otherwise.

At community level, the paper posits that communities are either acceptant or reluctant towards polygyny. Therefore, the effects of polygyny on IPV might vary across communities. Let’s consider *w*_*ij*_ a woman *i* in polygamous marriage living in a community *j*. If *n*_*j*_ denotes the total number of married or cohabiting women in a community (cluster) *j*, “community polygyny” is a quantity “*p*_*j*_” defined as follows:

1 $${p}_{j}=100* \frac{1}{{n}_{j}} \sum_{i=1}^{n}{w}_{ij}$$

This quantity ranges from 0 to 100. The higher (lower) this quantity, the more acceptant (reluctant) is the community towards polygyny. In this paper, community polygyny is treated as a continuous variable.

###### Other variables

There is an abundant literature on risk factors associated with IPV [46–48]. For instance, previous research showed that women who are more empowered educationally, economically and socially are usually most protected against IPV [46]. In this study, women’s education was captured in completed years. Other studies have focused on urban residence and showed how urban residence could explain the prevalence of IPV in SSA [49, 50]. A meta-analysis on IPV in SSA showed that women living in rural areas experienced higher rates of IPV than urban counterparts. In this study, place of residence was coded ‘1’ if the woman resided in urban areas, and ‘0’ otherwise. Poverty was also found to be associated with IPV: women in better-off households experienced lower rates of IPV [51, 52]. Poverty in this study was proxied by household wealth index (HWI). The methodology to derive HWI from DHSs data has been published elsewhere [53, 54]. HWI in the original dataset was categorized as ‘poorest’, ‘poorer’, ‘middle’, “richer’, and ‘richest’. In this study, the original variable of wealth quintile was recorded into three categories: Poor (bottom 40%) coded “1”; Middle (20%) coded 2 and Rich (top 40) coded “3”. The index of media exposure was created from three variables: the frequency of watching television, listening to radio, or reading newspapers/magazines. Responses to these variables were ‘0’ if respondent reported ‘not at all’, ‘1’ for ‘less than once a week’, and ‘2’ for ‘at least once a week’. Responses were recorded into ‘0 = No’ for ‘not at all’ and ‘1 = Yes’ for ‘less than once a week’ and ‘at least once a week’. A dichotomous variable was created from a composite of exposure to the three media sources and defined as “0 = No” for married/cohabiting women who scored ‘0’ on the three items and ‘1 = Yes’ if women’s score was higher or equal to ‘1’. Finally, attitudes towards violence were associated with IPV [27, 55–58]. The variable ‘justification of violence’ was derived from questions asking married women if it is justified for a husband to beat his wife for the following reasons: (*i*) burning food, (*ii*) arguing with him, (*iii*) going out without telling him, (*iv*) neglecting the children, and (*v*) refusing to have sexual intercourse with him. A binary variable was created from these five reasons to reflect the attitudes towards wife beating. Justification of violence was therefore coded as ‘0 = No’ if a woman disagreed with the five reasons and ‘1 = Yes’ if she agreed to at least one of these reasons.

###### Analytical strategy

##### Descriptive statistics

The paper begins with descriptive analyses which consisted in mapping the outcome (intimate partner violence) and the key independent variables: polygyny at women and community levels to unveil the heterogeneity of the outcome and key independent variables across provinces in each country. Indeed, previous studies showed that polygyny unevenly affects girls and women regarding their exposure to IPV. Studies found that the likelihood of IPV was higher among women in polygynous unions [26–28, 59, 60]; however, this heterogeneity remains understudied. This study extends the existing literature, accounting for the differential effects of polygyny on IPV in Central Africa.

##### Modeling strategy

For multivariate analyses, this paper utilizes multilevel modeling to investigate contextual effects and to quantify the effects of women’s sociodemographics and other selected variables (e.g., household wealth index, urban residence) on IPV. Since women from the same cluster are assumably alike because they share a common set of characteristics, this violates the standard assumption of independence of observations, which could produce downwardly biased variance estimates leading to false conclusions to inferring the existence of a significant association when there is no association with the outcome of interest. Furthermore, multilevel modeling allows to disentangle contextual from compositional effects by simultaneously modeling the effects of community- and individual-level predictors, with woman as unit of analysis [61]. Two-level logistic regression models were performed as follows, in which *i* and *j* refer to woman- and community-level variables, respectively:

2.a $$logit\left(\frac{{\pi }_{ij}}{{1-\pi }_{ij}}\right)= {\beta }_{0}+\sum_{k=1}^{p}{\beta }_{k}{x}_{ij}^{k}+\sum_{l=1}^{q}{\delta }_{l }{z}_{j}^{l}$$

2.b $${\beta }_{0j}={\beta }_{0}+{u}_{0j}$$

The quantity ${\pi }_{ij}$ is the probability that a sampled woman referenced (*i*, *j*) experienced IPV in the last 12 months preceding the survey; ${x}_{ij}^{k}$ and ${z}_{j}^{l}$ are the *k*^*th*^ woman-level covariates and *l*^*th*^ community polygyny respectively; ${\beta }_{0j}$ represents the interpret modelled to randomly vary across clusters; the estimates ${\beta }_{k}$ and ${\delta }_{l}$ represent the regression coefficients of individual- and community-level covariates respectively; and ${u}_{0j}$ is the random cluster residuals distributed as $N\left({0, \sigma }_{u}^{2}\right)$. The Eq. (2.a) can be extended to include cross-level interaction terms as follows [61, 62]:

2.c $$logit\left(\frac{{\pi }_{ij}}{{1-\pi }_{ij}}\right)= {\beta }_{0}+\sum_{k=1}^{p}{\beta }_{k}{x}_{ij}^{k}+\sum_{l=1}^{q}{\delta }_{l }{z}_{j}^{l}+ \sum_{k=1}^{r}\sum_{l=1}^{s}{\lambda }_{kl}{x}_{ij}^{k}{z}_{j}^{l}$$

In the Eq. (2.c), the estimated coefficients ${\lambda }_{kl}$ are the cross-level interaction terms. Specifically, this refers to the interactions between the community polygyny and (*i*) polygyny at women level; (*ii*) women’s education; and (*iii*) urban residence.

Analyses were performed using STATA SE version 18 for macOS, and accounting for the complex survey design (weighting, clustering, and stratification) to ensure that the estimates computed were unbiased and representative of the study population. Besides the null model (Model 0) allowing for a theoretical justification of multilevel modeling, five models were estimated. Model 1 included all individual-level covariates to obtain adjusted odd ratios (AOR). Model 2 extended Model 1 by including the key independent variable (community polygyny). Model 3 added random effects of the community polygyny to test the differential effects of polygyny across communities in the different countries. Models 4–6 extended Model 3 by testing interactions between (*a*) community polygyny and individual polygyny; (*b*) women’s education and community polygyny; (*c*) urban residence and community polygyny.

### Ethics statement

The DHS obtained ethical clearance from the recognised Ethical Review Committees/Institutional Review Boards of the respective countries, and the Institutional Review Board of ICF International (USA) before the surveys were conducted. Written informed consent was obtained from the women before participation. The authors of this paper sought and obtained permission from the DHS programme to use the data. The data were completely anonymized; therefore, the authors did not seek further ethical clearance before their use.

### Findings

#### Descriptive results

**Fig. 1:**
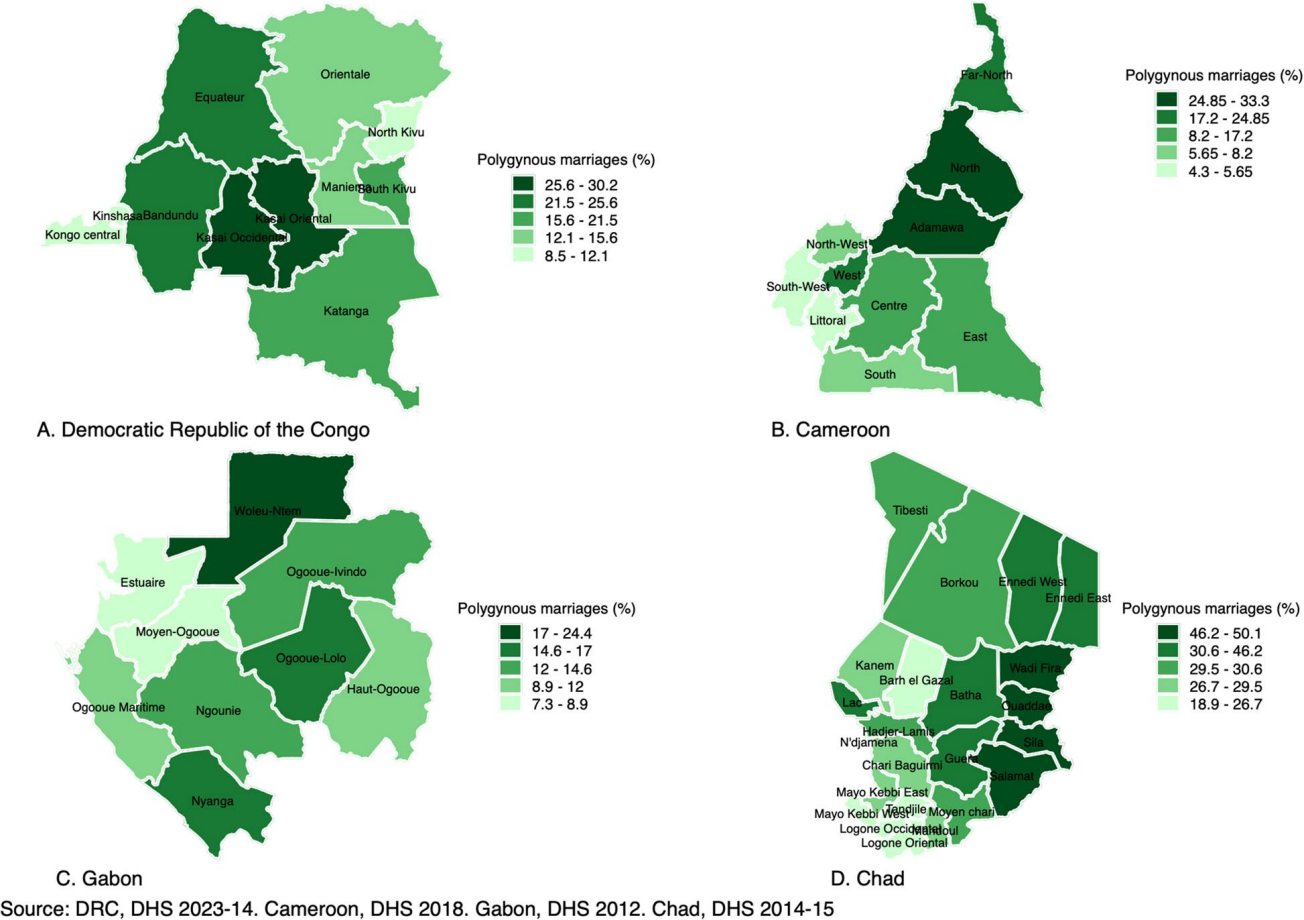
Percentage of married/cohabiting women of reproductive ages living in polygamous marriages

Table S.1 in appendix provides basic information regarding the sample sizes of married/cohabiting women included in the study for each country. The number of married/cohabiting included in the domestic violence module were 4,055 in Cameroon, 3,489 in Chad, 5,120 in the DRC, and 3,553 in Gabon. These numbers represent 50.3%, 26%, 41.1% and 74.8% of all women in union interviewed in each country, respectively. Also, women with missing information on the question “number of cowives” were excluded since it was not possible to determine whether they were living in monogamous or polygamous unions at the time of the survey. The percentages were marginal; they ranged from 0.8% in Chad to 7.6% in Gabon. Figure 1 below portrays the percentage of women in polygamous marriages/unions in each country.

The results showed significant variations of polygyny within and across countries (see Fig. 1; Panel A, B, C, D and Table S.3 in appendix; Column 2). At country level (Table S.2 in appendix), the percentage of women living in polygamous unions ranged from 4.3% in Cameroon to 50.1% in Chad. At provincial level, findings indicated that the percentage of women in polygamous unions was concentrated in the provinces of Kasai Occidental and Kasai Oriental with 30.2% and 27.3% in the Democratic Republic of the Congo (Fig. 1, Panel A), respectively. In Cameroon (Fig. 1, Panel B), percentage of women in polygamous unions was higher in the provinces of North and Far-North with 33.3% and 22.3%, respectively. In Gabon (Fig. 1, Panel C), the percentage of women living in polygamous unions was higher in the regions of Woleu-Ntem and Nyanga with 24.4% and 17.0%, respectively. In Chad (Fig. 1, Panel D), the prevalence was higher in the provinces of Ouaddae and Sila with 50.1% and 49.8% compared with the rest of country, respectively.

**Fig. 2:**
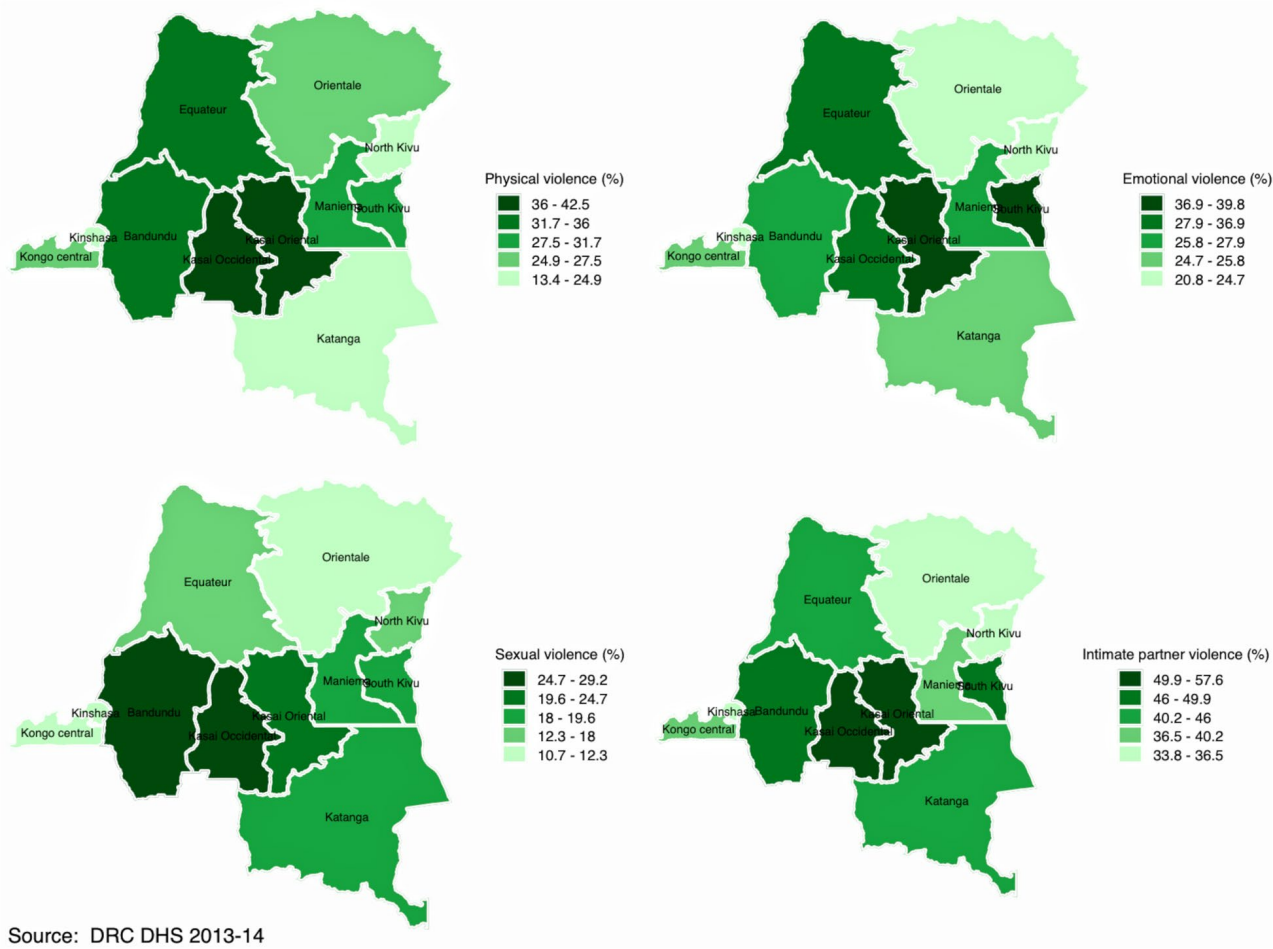
Percentage distribution of (**a**) physical violence; (**b**) emotional violence; (**c**) sexual violence; and (**d**) intimate partner violence among married/cohabiting women of reproductive ages in the Democratic Republic of the Congo

**Fig. 3:**
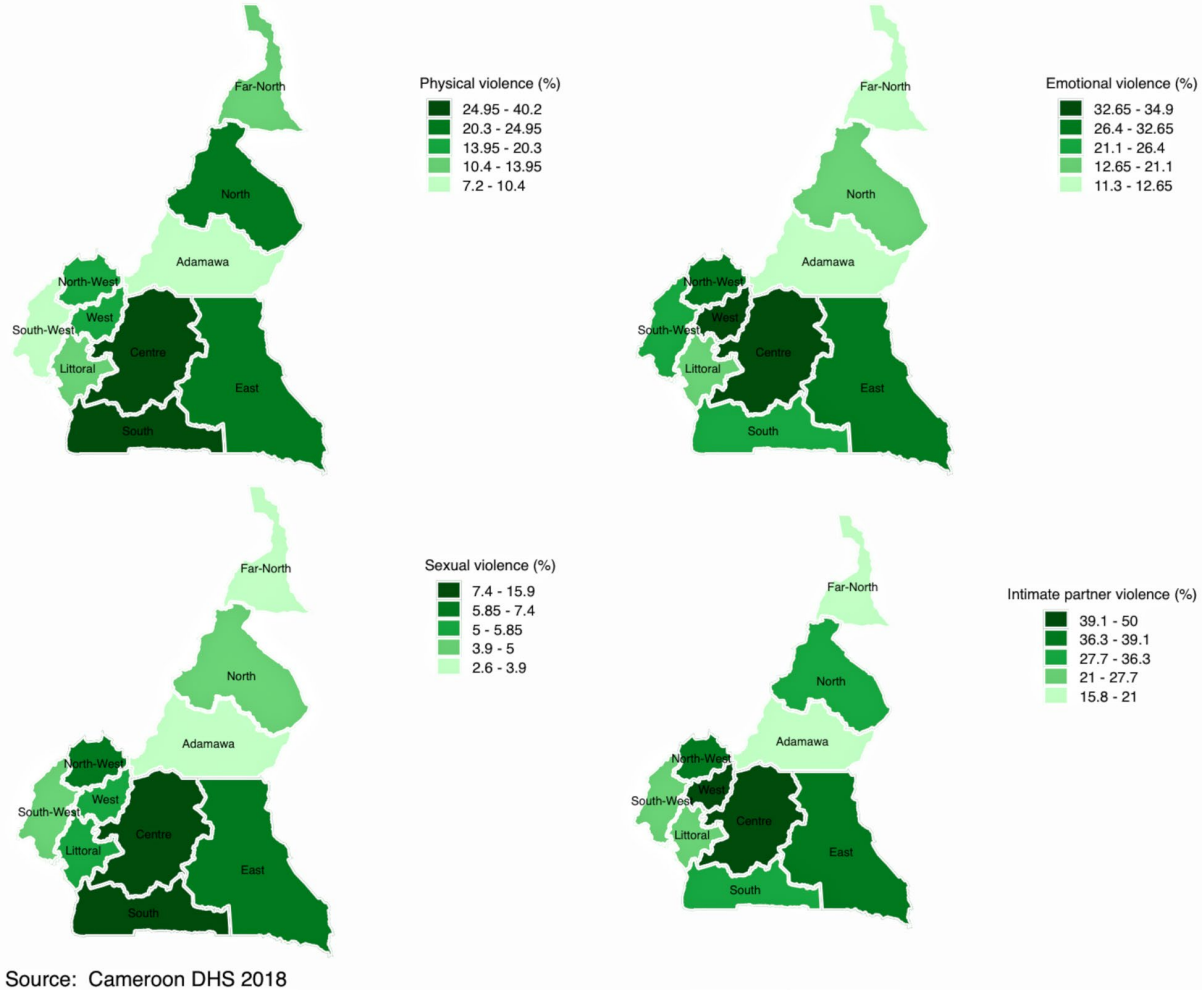
Percentage distribution of (**a**) physical violence; (**b**) emotional violence; (**c**) sexual violence; and (**d**) intimate partner violence among married/cohabiting women of reproductive ages in Cameroon

**Fig. 4:**
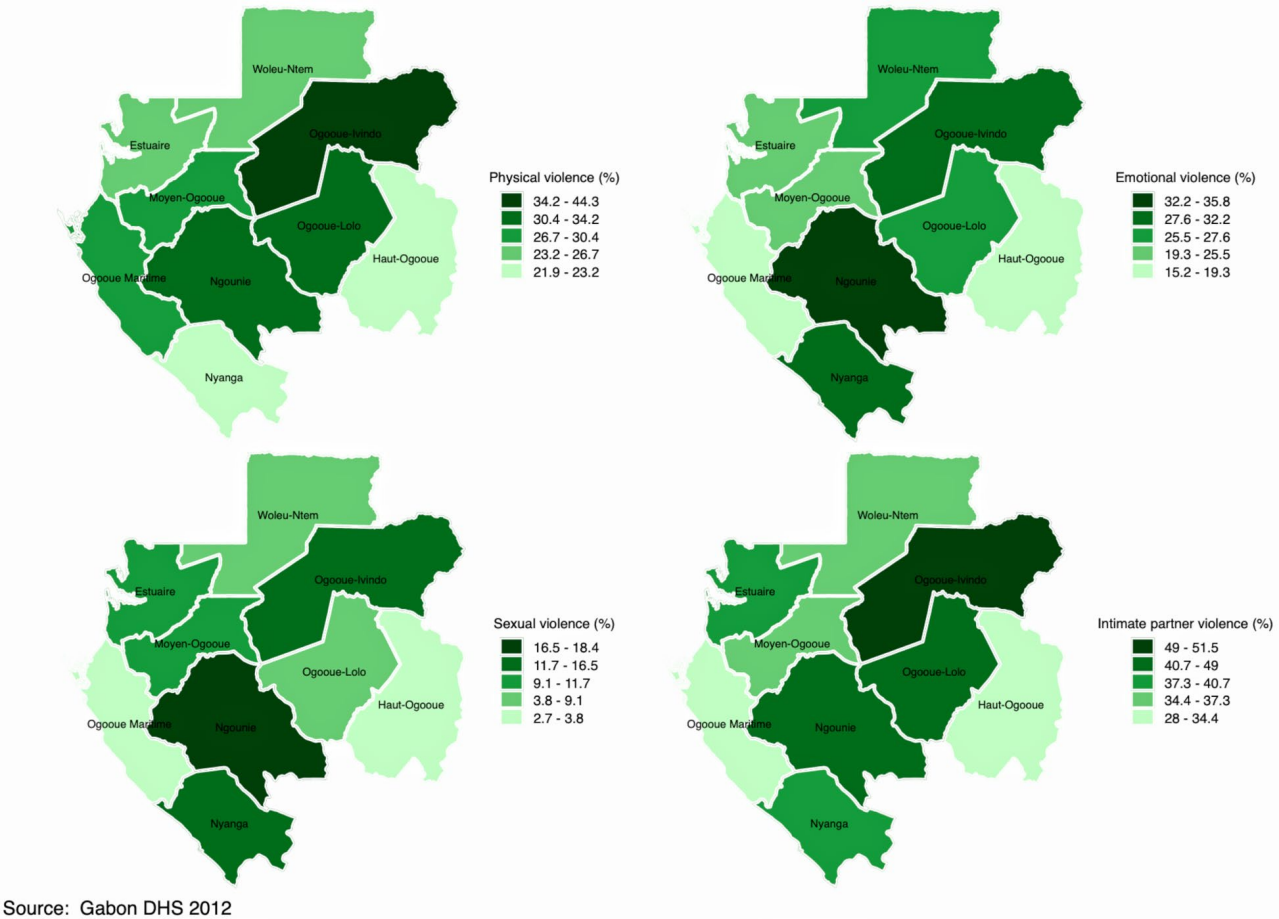
Percentage distribution of (**a**) physical violence; (**b**) emotional violence; (**c**) sexual violence; and (**d**) intimate partner violence among married/cohabiting women of reproductive ages in Gabon

**Fig. 5:**
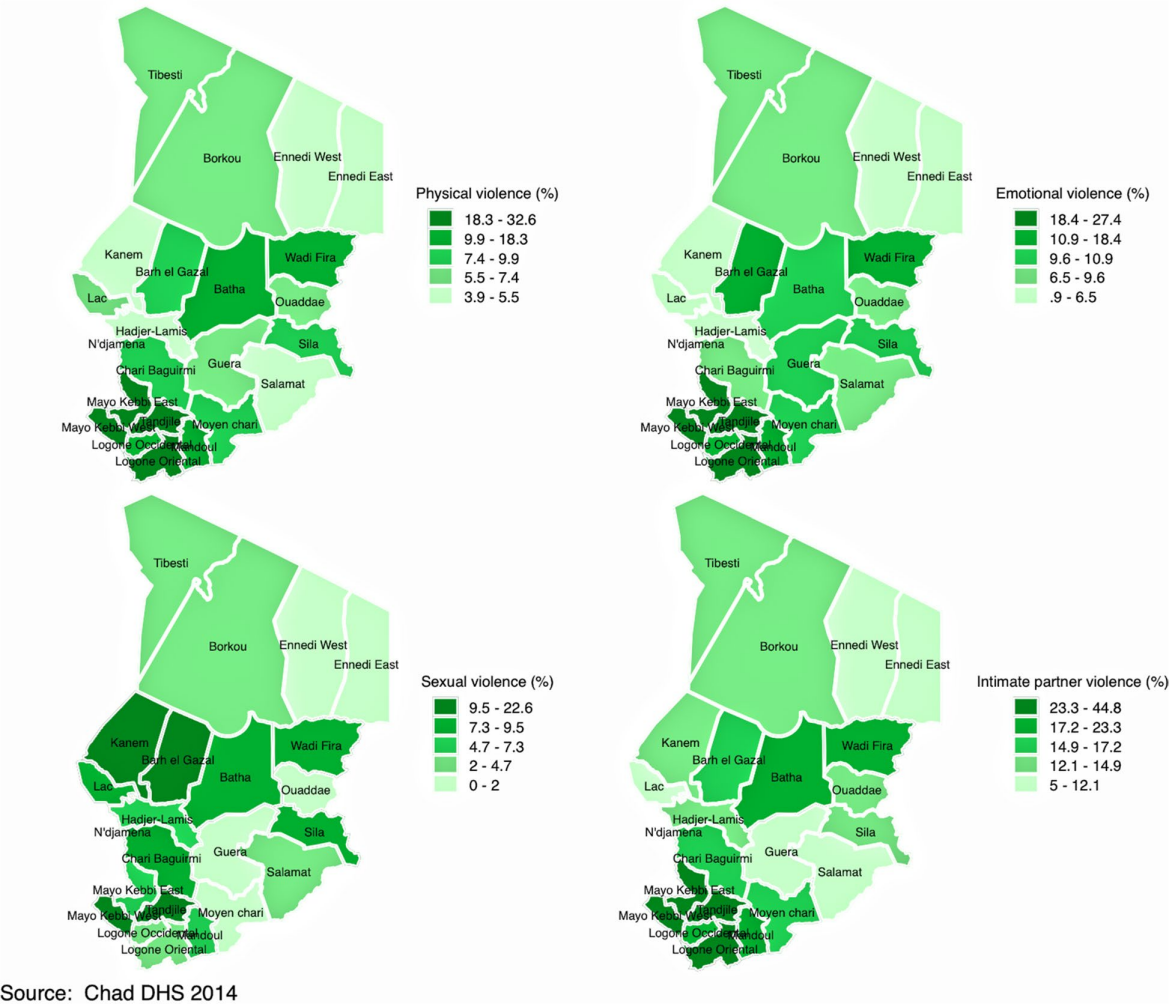
Percentage distribution of (**a**) physical violence; (**b**) emotional violence; (**c**) sexual violence; and (**d**) intimate partner violence among married/cohabiting women of reproductive ages in Chad

Figures 2, 3, 4 and 5 display spatial variations of IPV and its components (physical, emotional, and sexual violence). It should have been informative to decrypt each component of IPV; however, and for the sake of presentation, this section focuses on IPV and its variations within and across countries. In the DRC (Fig. 2), spatial distribution of IPV mimics the distribution of polygyny. Indeed, although the percentage of women who experienced IPV in the last 12 months varied from 33.8% in Kinshasa to 57.8% in Kasai Occidental, findings indicated that this percentage was higher in the provinces with higher percentage of polygamous unions, namely Kasai Occidental and Kasai Oriental. In Cameroon (Fig. 3), IPV prevalence ranged from 15.6% in Adamawa to 40.5% in the West. High prevalence of IPV was observed in the provinces of West (40.5%) and the Centre (38.8%). The Central province had the lowest prevalence of IPV (5.5%). In Gabon (Fig. 4), women who experienced IPV in the last 12 months ranged from 28% in Haut-Ogooue to 51.5% in Ogooue-Ivindo. In Chad (Fig. 5), the percentage of women who experienced IPV in the last 12 months ranged from 5% in Ennedi East and Ennedi West to 40% in Logone Oriental.

#### Multivariate results

The central thesis of the paper was to test the effects of community polygyny on IPV, assuming communities differ in their acceptance/reluctance levels towards polygyny, using multilevel modelling. Some communities are more acceptant while others are more reluctant based on the degree of the community polygyny. Tables 2, 3, 4, and 5 report estimated adjusted odds ratios (aOR) in the Democratic Republic of the Congo, Cameroon, Gabon, and Chad.

**Table 2:** Multilevel estimated odd ratios (OR) of intimate partner violence in the Democratic Republic of the Congo

Variables	Model 0	Model 1	Model 2	Model 3	Model 4	Model 5	Model 6
Fixed effects							
Polygynous unions (Ref.: Monogamous)		1.716***	1.624***	1.626***	1.906***	1.628***	1.625***
		(1.469—2.006)	(1.385—1.906)	(1.385—1.908)	(1.307—2.779)	(1.387—1.911)	(1.385—1.907)
Women's education (in single years)		0.985	0.986	0.985	0.985	0.974	0.986
		(0.965—1.005)	(0.966—1.006)	(0.966—1.006)	(0.966—1.006)	(0.941—1.009)	(0.966—1.006)
Urban residence (Ref.: Rural)		1.155	1.212	1.206	1.208	1.212	1.057
		(0.906—1.472)	(0.950—1.546)	(0.947—1.534)	(0.949—1.537)	(0.952—1.543)	(0.719—1.552)
Community polygyny			2.890***	2.983***	3.342***	2.320*	2.535**
			(1.461—5.719)	(1.474—6.039)	(1.587—7.039)	(0.902—5.968)	(1.146—5.607)
Interaction terms							
Polygynous unions X Community polygyny					0.579		
					(0.179—1.873)		
Women’s education X Community polygyny						1.054	
						(0.924—1.201)	
Urban residence X Community polygyny							2.033
							(0.405—10.212)
Random effects							
Random intercept (Variance)	0.732	0.614	0.596	0.508	0.514	0.508	0.510
	(0.580—0.923)	(0.476—0.792)	(0.461—0.771)	(0.343—0.752)	(0.347—0.760)	(0.344—0.752)	(0.344—0.754)
Random slope (Variance)				1.211	1.117	1.164	1.150
				(0.209—7.034)	(0.165—7.562)	(0.190—7.148)	(0.182—7.263)
Intraclass correlation (% ICC)	18.2	15.7	15.3	13.4	13.5	13.4	13.4
	(15.0—21.9)	(12.7—19.4)	(12.3—19.0)	(9.4—18.6)	(9.5—18.8)	(9.5—18.6)	(9.5—18.6)
Model Fit Statistics							
Akaike's information criterion (AIC)	6,784.35	6,518.39	6,511.13	6,511.56	6,512.73	6,512.95	6,512.82
Bayesian information criterion (BIC)	6,797.43	6,577.09	6,576.35	6,583.31	6,590.99	6,591.21	6,591.09
Observations	5,026	5,026	5,026	5,026	5,026	5,026	5,026
Number of groups	535	535	535	535	535	535	535

Statistical significance: *** *p* < 0.01, ** *p* < 0.05, * *p* < 0.1

Confidence intervals in parentheses

Model 1—6 controls for household wealth index, exposure to media, and attitude towards violence

**Table 3:** Multilevel estimated odd ratios (OR) of intimate partner violence in Cameroon

	**Model 0**	**Model 1**	**Model 2**	**Model 3**	**Model 4**	**Model 5**	**Model 6**
Variables							
Fixed effects							
Polygynous unions (Ref.: Monogamous)		0.894	0.992	0.998	1.433	1.013	0.999
		(0.717—1.114)	(0.790—1.245)	(0.793—1.255)	(0.931—2.206)	(0.805—1.275)	(0.795—1.256)
Women's education (in single years)		1.017	1.005	1.005	1.006	0.986	1.007
		(0.993—1.041)	(0.981—1.030)	(0.982—1.030)	(0.983—1.031)	(0.956—1.017)	(0.983—1.031)
Urban residence (Ref.: Rural)		0.793*	0.753**	0.717**	0.716**	0.727**	0.550***
		(0.609—1.031)	(0.579—0.978)	(0.555—0.926)	(0.555—0.925)	(0.564—0.938)	(0.400—0.757)
Community polygyny (Continuous)			0.278***	0.212***	0.252***	0.125***	0.110***
			(0.143—0.539)	(0.100—0.450)	(0.116—0.547)	(0.049—0.316)	(0.044—0.272)
Interaction terms							
Polygynous unions X Community polygyny					0.323*		
					(0.102—1.026)		
Women’s education X Community polygyny						1.126*	
						(0.998—1.270)	
Urban residence X Community polygyny							6.251***
							(1.619—24.133)
Random effects							
Random intercept (Variance)	0.549	0.521	0.511	0.308	0.301	0.311	0.303
	(0.403—0.749)	(0.371—0.734)	(0.364—0.717)	(0.167—0.570)	(0.162—0.560)	(0.171—0.568)	(0.164—0.559)
Random slope (Variance)				2.743	2.845	2.416	2.479
				(1.075—6.998)	(1.154—7.011)	(0.897—6.508)	(0.933—6.585)
Intraclass correlation (% ICC)	14.3	13.7	13.4	8.6	8.4	8.7	8.4
	(10.9–18.5)	(10.1–18.2)	(10.0—17.9)	(4.8–14.8)	(4.7—14.5)	(4.9—14.7)	(4.7—14.5)
Model Fit Statistics							
Akaike's information criterion (AIC)	4,872.23	4,655.17	4,642.47	4,636.34	4,634.66	4,634.66	4,631.34
Bayesian information criterion (BIC)	4,884.84	4,711.66	4,705.23	4,705.38	4,709.97	4,709.98	4,706.65
Observations	3,930	3,930	3,930	3,930	3,930	3,930	3,930
Number of groups	429	429	429	429	429	429	429

Statistical significance: *** *p* < 0.01, ** *p* < 0.05, * *p* < 0.1

Confidence intervals in parentheses

Model 1—6 controls for household wealth index, exposure to media, and attitude towards violence

**Table 4:** Multilevel estimated odd ratios (OR) of intimate partner violence in Gabon

Variables	Model 0	Model 1	Model 2	Model 3	Model 4	Model 5	Model 6
Fixed effects							
Polygynous unions (Ref.: Monogamous)		0.985	1.049	1.048	0.886	1.053	1.048
		(0.788—1.232)	(0.830—1.325)	(0.830—1.324)	(0.563—1.395)	(0.833—1.331)	(0.829—1.324)
Women's education (in single years)		1.009	1.009	1.009	1.009	1.036**	1.009
		(0.988—1.031)	(0.988—1.031)	(0.988—1.031)	(0.988—1.031)	(1.000—1.072)	(0.987—1.031)
Urban residence (Ref.: Rural)		1.040	1.038	1.037	1.044	1.045	0.960
		(0.846—1.280)	(0.844—1.275)	(0.844—1.275)	(0.849—1.284)	(0.849—1.286)	(0.715—1.289)
Community polygyny (Continuous)			0.504*	0.502*	0.435**	1.356	0.389*
			(0.237—1.074)	(0.233—1.080)	(0.189—0.999)	(0.371—4.961)	(0.136—1.109)
Interaction terms							
Polygynous unions X Community polygyny					2.084		
					(0.384—11.321)		
Women’s education X Community polygyny						0.852*	
						(0.719—1.010)	
Urban residence X Community polygyny							1.719
							(0.389—7.591)
Random effects							
Random intercept (Variance)	0.279	0.239	0.234	0.231	0.234	0.239	0.225
	(0.186—0.420)	(0.147—0.389)	(0.143—0.382)	(0.131—0.405)	(0.143—0.382)	(0.139—0.414)	(0.127—0.401)
Random slope (Variance)				0.097	0.000	0.006	0.196
				n.a	n.a	n.a	n.a
Intraclass correlation (% ICC)	7.8	6.8	6.6	6.5	6.6	6.8	6.4
	(5.3—11.3)	(4.3—10.6)	(4.2—10.4)	(3.8—10.9)	(4.2—10.4)	(4.0—11.1)	(3.7–10.9)
Model Fit Statistics							
Akaike's information criterion (AIC)	4,775.80	4,305.00	4,303.85	4,305.85	4,305.12	4,304.44	4,307.32
Bayesian information criterion (BIC)	4,788.15	4,359.90	4,364.84	4,372.93	4,372.22	4,377.63	4,380.51
Observations	3,293	3,293	3,293	3,293	3,293	3,293	3,293
Number of groups	334	334	334	334	334	334	334

Statistical significance: *** *p* < 0.01, ** *p* < 0.05, * *p* < 0.1

Confidence intervals in parentheses

Model 1—6 controls for household wealth index, exposure to media, and attitude towards violence

**Table 5:** Multilevel estimated odd ratios (OR) of intimate partner violence in Chad

Variables	Model 0	Model 1	Model 2	Model 3	Model 4	Model 5	Model 6
Fixed effects							
Polygynous unions (Ref.: Monogamous)		1.305**	1.327***	1.327***	1.564	1.308**	1.329***
		(1.060—1.605)	(1.072—1.642)	(1.072—1.642)	(0.874—2.797)	(1.056—1.620)	(1.073—1.644)
Women's education (in single years)		1.043**	1.043**	1.043**	1.042**	1.148***	1.041**
		(1.006—1.082)	(1.005—1.081)	(1.005—1.081)	(1.005—1.081)	(1.056—1.247)	(1.004—1.080)
Urban residence (Ref.: Rural)		1.151	1.148	1.148	1.149	1.103	1.606
		(0.799—1.658)	(0.797—1.653)	(0.797—1.653)	(0.798—1.656)	(0.765—1.592)	(0.686—3.757)
Community polygyny (Continuous)			0.749	0.749	0.875	1.173	0.900
			(0.319—1.763)	(0.319—1.763)	(0.323—2.371)	(0.466—2.954)	(0.347—2.336)
Interaction terms							
Polygynous unions X Community polygyny					0.667		
					(0.176—2.528)		
Women’s education X Community polygyny						0.753**	
						(0.602—0.941)	
Urban residence X Community polygyny							0.407
							(0.052—3.198)
Random effects							
Random intercept (Variance)	1.374	1.227	1.221	1.221	1.228	1.219	1.223
	(1.024–1.844)	(0.898—1.676)	(0.893—1.670)	(0.893—1.670)	(0.898—1.679)	(0.891—1.667)	(0.895—1.672)
Random slope (Variance)				0.000	0.000	0.000	0.000
				na	na	na	Na
Intraclass correlation (% ICC)	29.5	27.2	27.1	27.0	27.1	27.0	27.1
	(23.7 −35.9)	(21.4—33.8)	(21.4—33.7)	(21.4 −33.7)	(21.4–33.8)	(21.3–33.6)	(21.4–33.7)
Model Fit Statistics							
Akaike's information criterion (AIC)	3243.05	3183.14	3184.70	3184.70	3186.35	3180.36	3185.97
Bayesian information criterion (BIC)	3255.36	3238.45	3246.16	3246.16	3253.95	3247.96	3253.57
Observations	3,489	3,449	3,449	3,449	3,449	3,449	3,449
Number of groups	615	614	614	614	614	614	614

Statistical significance: *** *p* < 0.01, ** *p* < 0.05, * *p* < 0.1

Confidence intervals in parentheses

Model 1—6 controls for household wealth index, exposure to media, and attitude towards violence

Models 0—3 in Tables 2, 3, 4 and 5 present the results of additive models. The results in Model 0 showed that a sizeable percentage of variance in intimate partner violence (IPV) was attributable to differences in characteristics of clusters. These percentage of variance ranged from 7.8% in Gabon to 29.5% in Chad. At individual level, the effects of polygyny, women’s education and urban residence varied across countries and not all these variables reached statistical significance. For instance, in the DRC, findings in Model 1 (Table 2) showed that living in polygamous unions increased the likelihood of IPV in the last 12 months by 72% (AOR = 1.716; 95% CI: 1.469—2.006). In Cameroon (Model 1; Table 3), urban residence decreased likelihood of suffering from IPV in the last 12 months by 21% (AOR = 0.793; 95% CI: 0.609—1.031). In Gabon, none of the variables reached statistical significance. In Chad, living in polygamous unions increased the likelihood of IPV by 30% (AOR = 1.305; 95% CI: 1.060—1.605). An additional year of women’s education increased the likelihood of IPV by 4.3% (AOR = 1.043; 95% CI: 1.006—1.082).

#### Multiplicative models

Models 4—6 (multiplicative models) in Tables 2, 3, 4 and 5 present the interactive effects of community polygyny and (*a*) individual polygyny (Model 4); (*b*) women’s education (Model 5); and (*c*) urban residence (Model 6). Of chief importance in the present study is the investigation of potential interactions whereby the women’s polygyny and community polygyny interact to produce substantively different levels of IPV, or specifically, the extent to which community polygyny moderate, exacerbate or mitigate the estimated effects of individual women’s polygyny on IPV. The results showed that the effects of women’s polygyny and community polygyny on IPV varied across countries. Although the interaction terms did not reach statistical significance, the results showed that community polygyny mitigates the effects of women’s polygyny in all countries except Gabon. Second, the interaction between women’s education and community polygyny was introduced (Model 6, Tables 2, 3, 4 and 5). The pattern is different for each country. While community polygyny and women’s education interact to exacerbate IPV in the DRC and Cameroon, it mitigates IPV in Gabon and Chad. Third, interactions between urban residence and community polygyny tends to exacerbate IPV in three countries, namely the DRC, Cameroon, and Gabon.

### Discussion and conclusion

The paper examined the differential effects of community polygyny on IPV in Central Africa. More specifically, this paper extended existing literature at individual level on the negative effects of polygyny and IPV [26, 63, 64]. Most studies showed that women in polygamous unions were more likely to experience IPV in the last 12 months compared with those in monogamous unions. These studies are surely informative and have provided insights to further our understanding between polygyny and IPV. The current study added another dimension by conceptualizing and defining “community polygyny” and estimating its effects on IPV. To the best of our knowledge, this is the first study to investigate the effect of polygyny on IPV at community level. The basic assumption is that some communities accept polygyny while others do not. In the following section, the paper focuses on the main effects (Model 3) and interactive effects of community polygyny on IPV.

Our analyses suggest a differential effects of community polygyny on IPV in the four countries. In the DRC, community polygyny significantly increased the likelihood of IPV, while it decreased the likelihood of IPV in Cameroon, Gabon, and Chad. These findings reinforce the need of context-specific studies on health and socioeconomic outcomes in sub-Saharan Africa [65]. There are increasingly studies in SSA pooling data from different countries and estimating the associations between outcomes and independent variables [66, 67]. These studies have the advantage of increasing statistical power [50, 68–71]. However, in doing so, these studies ignore the heterogeneity (or diversity) of countries, and such findings can be misleading in a programmatic point of view. In fact, not only countries (and therefore contexts) are different, but even within countries, patterns could be different. The descriptive results clearly showed diversity: polygyny and IPV were not equally distributed at individual, community, and provincial/regional levels in selected countries. For instance, the prevalence of polygamous unions in the DRC was higher in Kasai Occidental and Kasai Oriental, and that of IPV mimicked the prevalence of polygyny. In contrast, while polygamous unions were higher in Chad compared with other countries, that was not the case for the prevalence of IPV. Such findings call for more context-specific studies rather than pooling data to overlook diversities within and across countries.

### Interactive effects

This study tested the interactions between community polygyny and (*a*) women’s polygyny; (*b*) women’s education; and (*c*) urban residence. Previous studies showed that women in polygamous unions were more likely to experience IPV in the last 12 months preceding the survey [26–29, 72, 73]. There are plausible theoretical foundations regarding the interactions tested in this study. First, in communities where polygyny is more accepted, women would be more likely to normalize polygamous unions. In this case, it is expected that community polygyny would have either null or negative effects on IPV. Since there is no information regarding the “acceptance” of polygyny in a community, the paper assumed that the higher the prevalence of polygyny, the more acceptant is the community. Second, women’s education is generally considered an asset for social/health outcomes, including IPV. More educated are less likely to be exposed to IPV (for a thorough discussion, see [48]). In the context of SSA, there might be competing hypotheses regarding the effects of women’s education and IPV, as well as its interaction with community polygyny. Since education might be an indication of communication openness with husband/partner which empowers women in negotiations (e.g., sexual relationships), it might also be a source of tensions in the couples if the husband/partner is less willing to comprehend his wife/partner. Finally, urban residence has been associated with positive social/health outcomes. It was expected urban residence to be associated with lower risks of IPV, and a mitigating effect between urban residence and community polygyny. Overall, the interactive effects between community polygyny and the three key independent variables (polygyny, education, and urban residence) were less conclusive. Therefore, more research is needed to unveil the joint effects of community polygyny and (*a*) women’s polygyny; (*b*) education; and (*c*) urban residence.

Programmatically, findings stressed the importance to accounting for acceptance levels of polygyny within communities to design more effective IPV-related interventions to ensure that (*a*) more disadvantaged communities are better served; and (*b*) elimination of violence against women and girls in Central Africa could be achieved according to SDG agenda.

### Strengths and limitations of the study

The study used nationally representative surveys to study the associations between three key variables (women’s polygyny, education, and urban residence), community polygyny and IPV in Central Africa. The paper reinforced the needs of context-specific studies to further our understanding of social and health outcomes in Central Africa, where socioeconomic indicators are worst compared with other sub-regions in SSA [43] and which recorded the highest IPV prevalence in SSA [50]. The study has however some limitations. First, excluding some women of reproductive ages led to a loss of statistical power. Second, the study is based on self-reported IPV. This could be problematic due to cultural norms to hiding violence from husbands/partners. Finally, the 12-month period might lead to recall bias and therefore might underestimate IPV prevalence in the four selected countries in Central Africa.

## Supplementary Information

Supplementary Material 1.

## Data Availability

No datasets were generated or analysed during the current study.
