# Supplementary material for: Measuring the effects of community polygyny on intimate partner violence: a multilevel modeling using nationally representative cross-sectional data
Source: Reprod Health. 2025 May 29;22:93. doi: 10.1186/s12978-025-02037-7 (PMC12121072; doi:10.1186/s12978-025-02037-7)
Supplement: Supplementary file 1 — Supplementary Material 1. [file 12978_2025_2037_MOESM1_ESM.zip › SI/Table S1.docx]

**Table S.1:**

**Number of women in union and number of women in domestic violence module by country in Central Africa**

| Number of women in the study | Country | | | |
| --- | --- | --- | --- | --- |
|  | Cameroon | Chad | Democratic  Republic of the Congo | Gabon |
| Number of married/cohabiting women | 8,060 | 13,439 | 12,448 | 4,749 |
| Number of married/cohabiting women in domestic violence module | 4,055 (50.3%) | 3,489 (26.0%) | 5,120  (41.1%) | 3,553 (74.8%) |
| Number of married/cohabiting women in domestic violence module missing information about cowives | 256  (3.2%) | 112 (0.8%) | 260  (2.1%) | 359  (7.6%) |

**Table S.2:**

**Percentage of women in union in domestic violence module living in polygamous unions by country**

| **Estimates** | **Country** | | | |
| --- | --- | --- | --- | --- |
|  | Cameroon | Chad | Democratic  Republic of the Congo | Gabon |
| Number of provinces/regions | 10 | 23 | 11 | 9 |
| Percentage of women in polygamous unions | 14.6 | 34.3 | 18.6 | 13.9 |
| Minimum | 4.3 | 18.9 | 8.5 | 7.3 |
| Maximum | 33.3 | 50.1 | 30.2 | 24.4 |

**Table S.3:**

Prevalence of (1) polygamous marriages; (2) physical violence; (3) emotional violence; (4) sexual violence; and (5) intimate partner violence in Central Africa

| **A. Democratic Republic of the Congo** |  |  |  |  |  |
| --- | --- | --- | --- | --- | --- |
| Province/Region | **(1)** | **(2)** | **(3)** | **(4)** | **(5)** |
| Kinshasa | 8.9 | 21.0 | 20.8 | 10.7 | 33.8 |
| Bandundu | 22.6 | 34.0 | 27.2 | 25.5 | 47.7 |
| Kongo Central | 12.1 | 27.5 | 25.8 | 11.5 | 37.3 |
| Equateur | 25.6 | 36.0 | 29.6 | 18.0 | 46.0 |
| Kasai Occidental | 30.2 | 42.5 | 36.9 | 29.2 | 57.6 |
| Kasai Oriental | 27.3 | 36.4 | 38.7 | 24.7 | 53.3 |
| Katanga | 17.5 | 24.9 | 25.2 | 18.5 | 40.8 |
| Maniema | 15.0 | 29.7 | 27.9 | 19.6 | 40.2 |
| North Kivu | 8.5 | 13.4 | 24.7 | 17.6 | 36.5 |
| Orientale | 15.6 | 26.2 | 23.0 | 12.3 | 35.8 |
| South Kivu | 21.5 | 31.7 | 39.8 | 19.6 | 49.9 |
| Total | 18.6 | 29.4 | 29.1 | 18.8 | 43.5 |
| **B. Cameroon** | | | | | |
| Adamawa | 27.4 | 7.2 | 11.3 | 3.3 | 15.8 |
| Centre | 5.5 | 28.8 | 26.2 | 11.5 | 38.8 |
| Littoral | 5.5 | 15.7 | 18.9 | 8.6 | 29.1 |
| East | 12.9 | 23.1 | 26.5 | 6.1 | 36.7 |
| Far-North | 22.3 | 10.8 | 11.8 | 2.6 | 17.6 |
| North | 33.3 | 24.8 | 19.6 | 4.5 | 30.1 |
| North-West | 7.7 | 17.5 | 31.8 | 6.8 | 37.7 |
| West | 21.5 | 14.3 | 34.9 | 5.6 | 40.5 |
| South | 5.8 | 25.1 | 26.3 | 8.0 | 35.9 |
| South-West | 4.3 | 10.0 | 22.6 | 4.6 | 25.3 |
| Total | 14.6 | 17.7 | 23.0 | 6.2 | 30.8 |
| **C. Gabon** | | | | | |
| Estuaire | 8.9 | 26.7 | 24.2 | 10.6 | 38.1 |
| Haut-Ogooue | 10.9 | 21.9 | 15.2 | 3.8 | 28.0 |
| Moyen-Ogooue | 7.3 | 27.9 | 25.5 | 11.7 | 37.3 |
| Ngounie | 14.6 | 33.2 | 35.8 | 18.4 | 48.0 |
| Nyanga | 17.0 | 23.2 | 30.4 | 16.5 | 40.7 |
| Ogooue Maritime | 12.0 | 30.4 | 19.3 | 2.7 | 34.4 |
| Ogooue-Ivindo | 14.3 | 44.3 | 32.2 | 13.5 | 51.5 |
| Ogooue-Lolo | 16.1 | 34.2 | 27.1 | 9.1 | 49.0 |
| Woleu-Ntem | 24.4 | 26.2 | 27.6 | 7.4 | 36.1 |
| Total | 13.9 | 29.8 | 26.4 | 10.4 | 40.3 |
| **D. Chad** | | | | | |
| Batha | 34.7 | 11.9 | 10.8 | 9.5 | 19.3 |
| Borkou | 30.6 | 6.9 | 8.0 | 3.7 | 14.9 |
| Tibesti | 30.6 | 6.9 | 8.0 | 3.7 | 14.9 |
| Chari Baguirmi | 26.8 | 8.0 | 9.6 | 8.9 | 16.3 |
| Guera | 46.2 | 5.6 | 10.9 | 0.7 | 12.1 |
| Hadjer-Lamis | 30.4 | 4.9 | 6.5 | 5.5 | 13.5 |
| Kanem | 27.5 | 5.2 | 5.7 | 11.9 | 14.4 |
| Lac | 39.3 | 5.9 | 3.4 | 8.4 | 10.1 |
| Logone Occidental | 18.9 | 18.3 | 18.4 | 2.1 | 22.2 |
| Logone Oriental | 26.1 | 28.6 | 25.9 | 4.7 | 40.0 |
| Mandoul | 29.3 | 14.6 | 16.3 | 5.5 | 23.3 |
| Mayo Kebbi East | 29.5 | 23.3 | 21.6 | 7.3 | 30.6 |
| Mayo Kebbi West | 25.6 | 32.6 | 23.4 | 22.6 | 44.8 |
| Moyen chari | 29.6 | 9.4 | 10.3 | 0.0 | 16.1 |
| Ouaddae | 50.1 | 7.4 | 8.4 | 0.8 | 12.8 |
| Salamat | 48.2 | 5.5 | 7.7 | 3.6 | 11.6 |
| Tandjile | 23.8 | 27.7 | 27.4 | 11.9 | 39.4 |
| Wadi Fira | 49.1 | 13.5 | 17.8 | 9.3 | 19.5 |
| N'djamena | 28.0 | 12.3 | 14.3 | 6.7 | 18.2 |
| Barh el Gazal | 26.7 | 9.9 | 11.5 | 13.1 | 17.2 |
| Ennedi East | 43.7 | 3.9 | 0.9 | 2.0 | 5.0 |
| Ennedi West | 43.7 | 3.9 | 0.9 | 2.0 | 5.0 |
| Sila | 49.8 | 9.1 | 10.5 | 8.7 | 13.6 |
| Total | 34.3 | 12.0 | 12.1 | 6.6 | 18.9 |

**Figures in Appendix: Captions**

Figure A.1:

Relationships between polygyny and intimate partner violence at regional/provincial level in Central Africa
