# Supplementary figures and images for: Measuring the effects of community polygyny on intimate partner violence: a multilevel modeling using nationally representative cross-sectional data
Source: Reprod Health. 2025 May 29;22:93. doi: 10.1186/s12978-025-02037-7 (PMC12121072; doi:10.1186/s12978-025-02037-7)

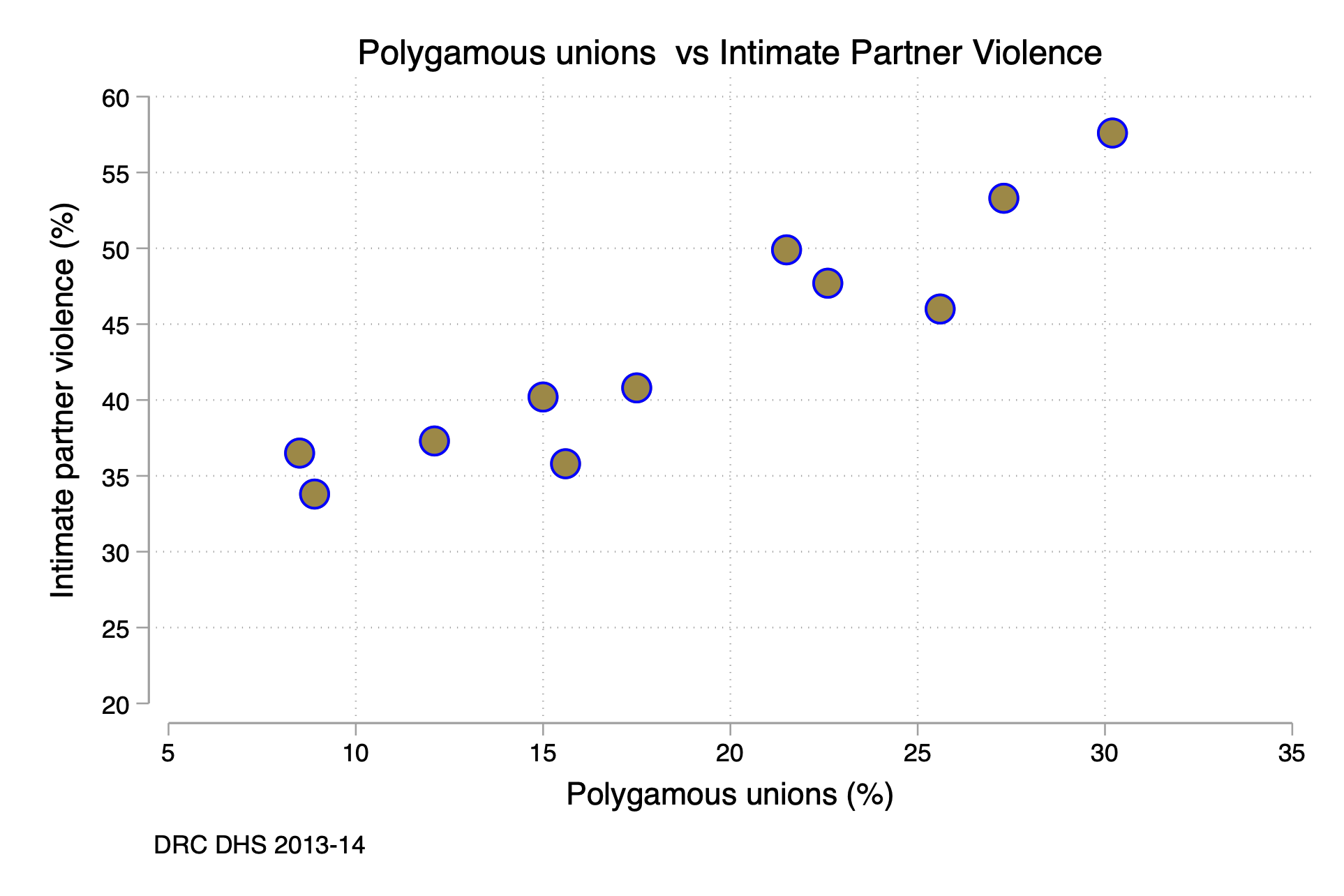

Supplement: Supplementary file 1 — Supplementary Material 1. [file 12978_2025_2037_MOESM1_ESM.zip › SI/Fig A.1.tif]

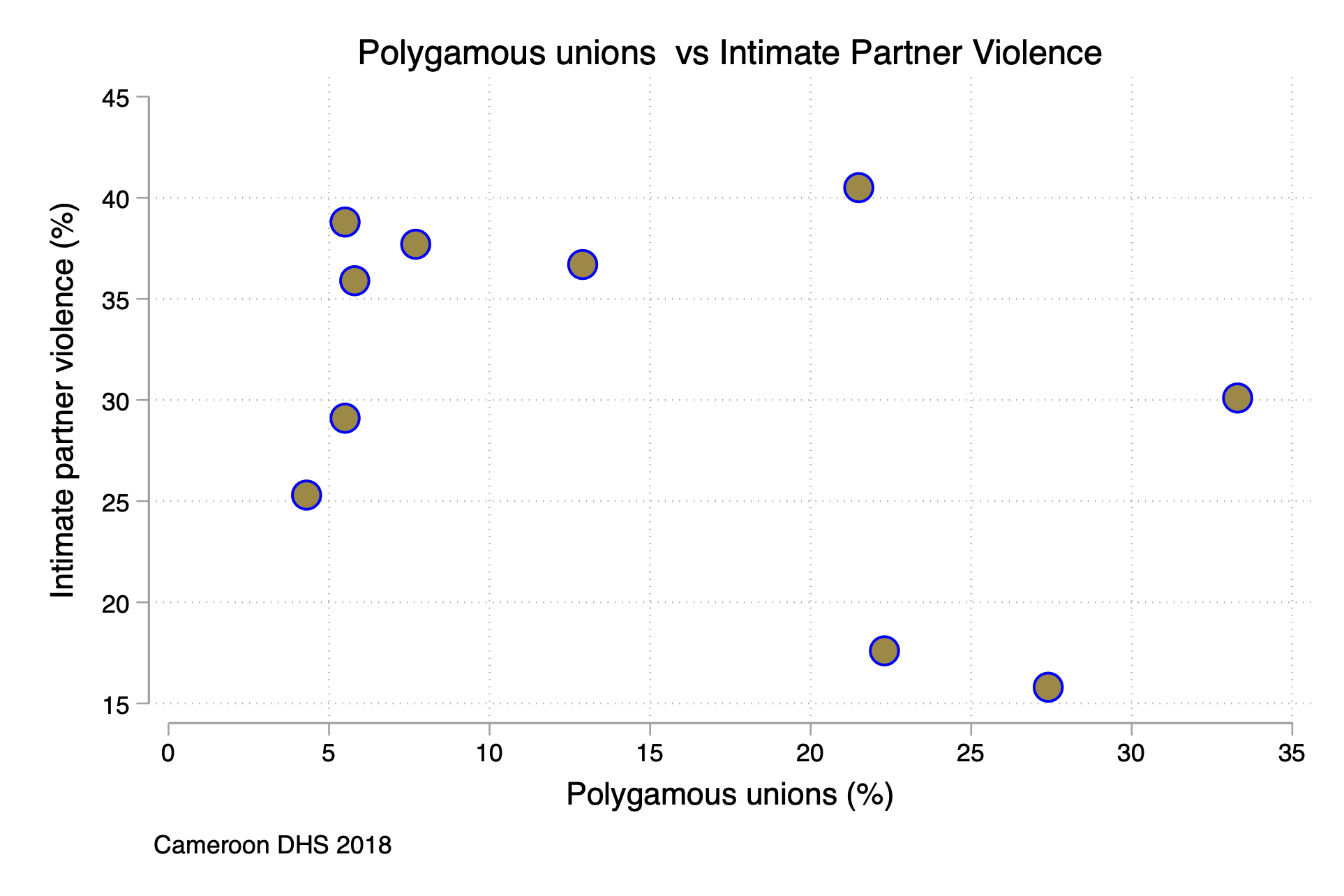

Supplement: Supplementary file 1 — Supplementary Material 1. [file 12978_2025_2037_MOESM1_ESM.zip › SI/Fig A.2.tif]

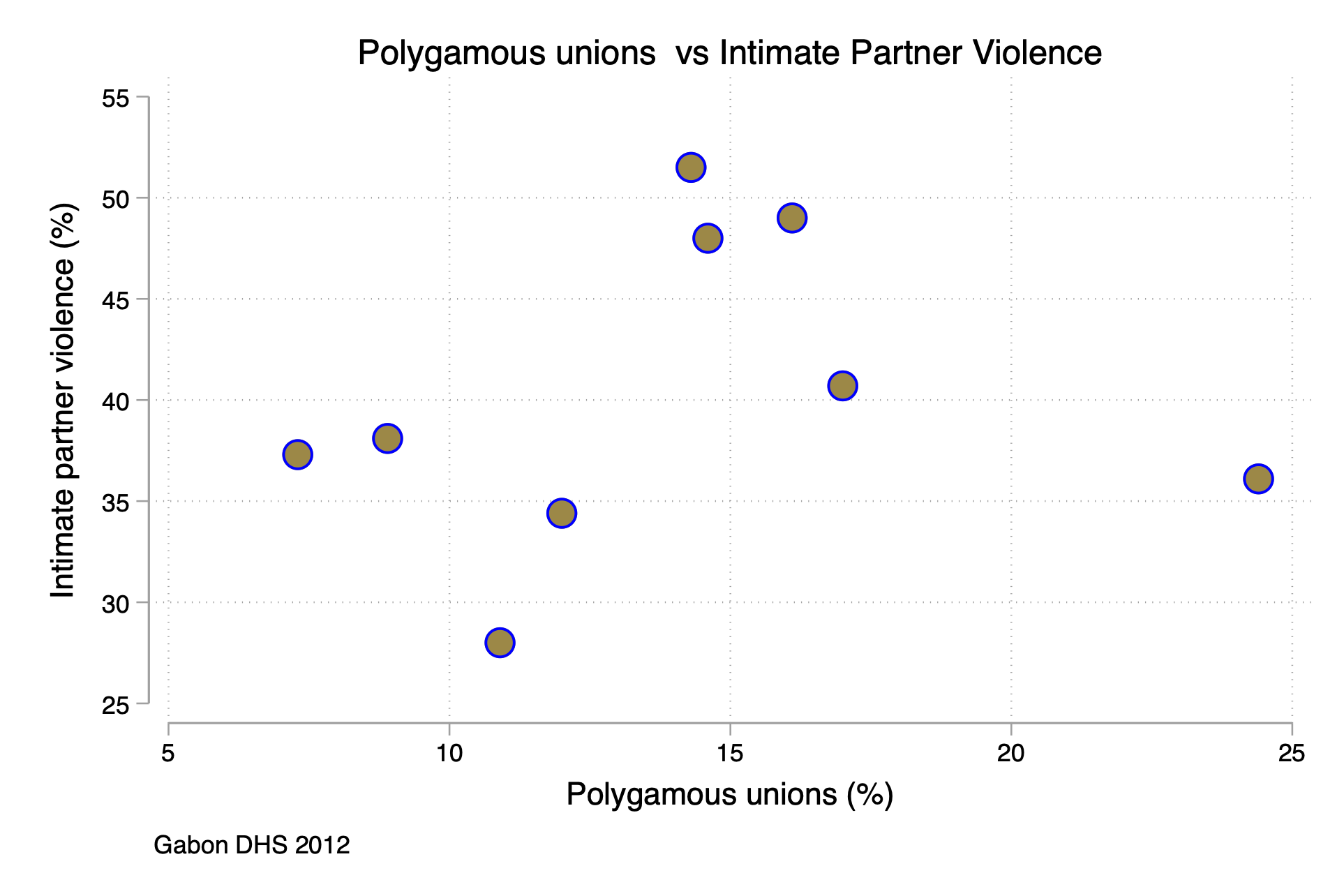

Supplement: Supplementary file 1 — Supplementary Material 1. [file 12978_2025_2037_MOESM1_ESM.zip › SI/Fig A.3.tif]

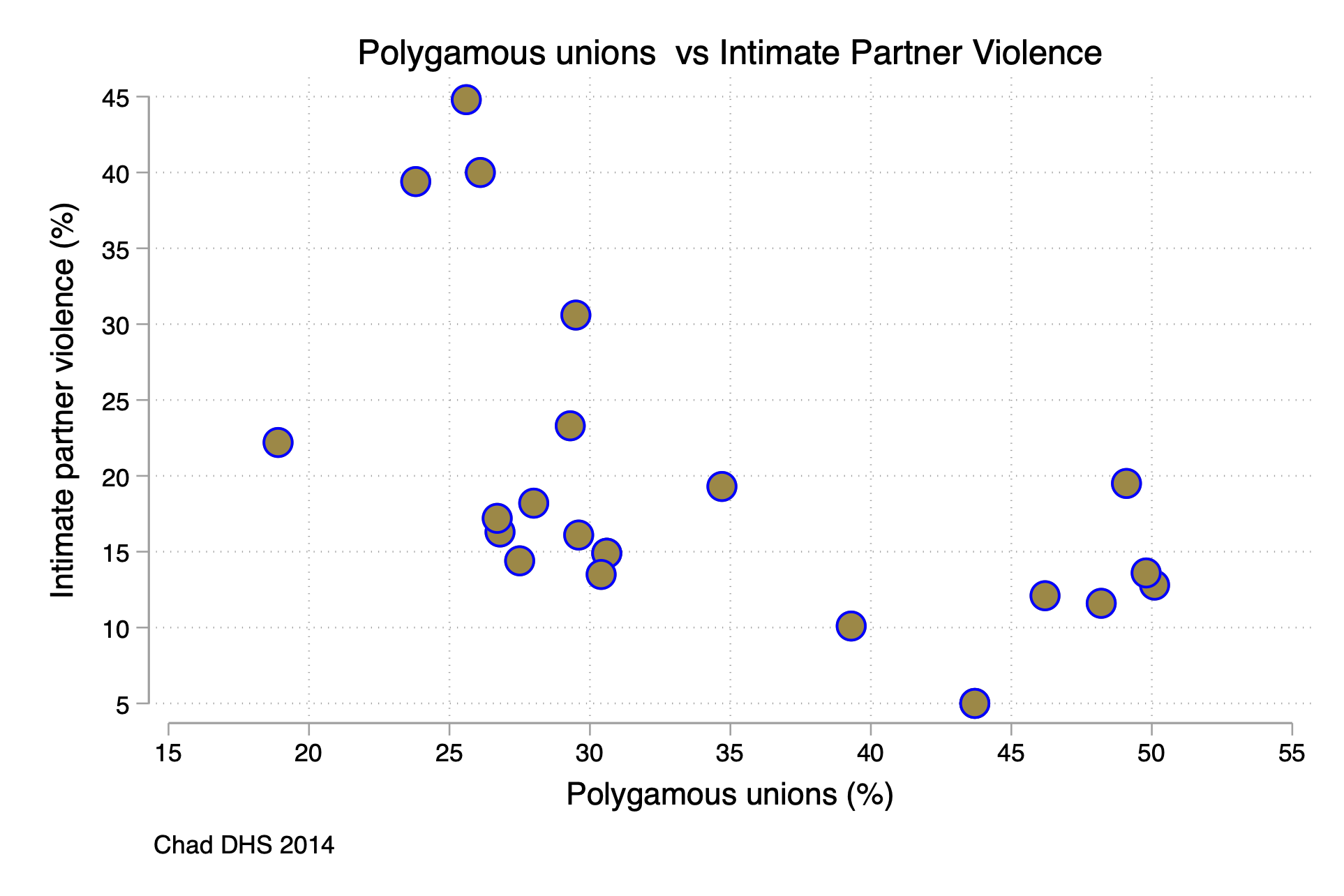

Supplement: Supplementary file 1 — Supplementary Material 1. [file 12978_2025_2037_MOESM1_ESM.zip › SI/Fig A.4.tif]
